# Visual inspection of vaccine storage conditions in general practices: A study of 75 vaccine refrigerators

**DOI:** 10.1371/journal.pone.0225764

**Published:** 2019-12-03

**Authors:** Anika Thielmann, Marie-Therese Puth, Birgitta Weltermann

**Affiliations:** 1 Institute for General Practice, University of Duisburg-Essen, University Hospital Essen, Essen, Germany; 2 Institute of General Practice and Family Medicine, University of Bonn, Bonn, Germany; 3 Department of Medical Biometry, Informatics and Epidemiology, Faculty of Medicine, University of Bonn, Bonn, Germany; All India Institute of Medical Sciences Bhopal, INDIA

## Abstract

**Introduction:**

Adequate vaccine storage is a prerequisite to assure vaccine effectiveness and tolerability. In this context, maintaining the cold chain (2°C to 8°C) is the paramount objective. To establish quality-ensured cold chain maintenance, compliance with several structural and procedural aspects is necessary.

**Main objective:**

The aim of this publication is to assess the quality of vaccine refrigerator management in general practices.

**Methods:**

This study describes baseline results of an intervention study. To evaluate the quality of vaccine refrigerator management, visual inspections were conducted of refrigerators used to store vaccines in general practices of a German teaching practice network. The study instrument was a checklist with ten quality criteria based on international best practices for vaccine storage. A data logger recorded refrigerator temperatures for 7 days. We analyzed associations between reaching more than half (6+) of the ten quality criteria and temperature data.

**Results:**

The study included 64 of 168 practices (38.1% response rate) with 75 refrigerators. No practice fulfilled all 10 quality criteria. On average, 4.7 (standard deviation = 1.9) criteria were met. The most frequent deficits were: no drawers/bins/baskets for vaccines (81.3%), no temperature logbook near refrigerator (75.0%), no temperature recording device in the center of the refrigerator (54.0%), vaccines boxes with contact to outer walls (46.3%), and refrigerator unsuitable for vaccine storage (44.6%). Refrigerators with better management (≥6 quality criteria) were more likely to have temperatures in the target range (62.5% vs. 27.5%, p = 0.008).

**Conclusion:**

We identified a large number of avoidable vaccine storage errors. Effective strategies, e.g. web-based programs, to improve vaccine storage conditions in general practices are needed.

## Introduction

Adequate vaccine storage is a prerequisite to assure the effectiveness and tolerability of vaccines [1,2]. Maintaining a continuous cold chain between +2 and +8°C is of utmost importance as the thermostability of vaccines varies considerably [1]. Freeze exposure is especially dangerous to adsorbed vaccines (e.g. hepatitis A, hepatitis B, tetanus, diphtheria, pertussis, pneumococcal disease), as the aluminum-containing adsorbents form irreversible precipitates which decrease vaccine potency and may induce local irritation upon injection [1,2]. Adsorbed hepatitis B vaccines are considered the most sensitive vaccines, with a freezing threshold of -0.5°C [1]. Also, heat exposure has a negative cumulative effect on vaccine potency [1]. For instance, pertussis vaccine is stable for 2 weeks at 20–25°C, for one week at 37°C, and loses 10% or more per day at >45°C [1]. Overall, the World Health Organization (WHO) considers protecting vaccines from freeze damage “one of the most poorly addressed problems in vaccine management” that requires attention in order to not jeopardize disease-prevention goals [3].

A recent systematic review focusing on freezing temperatures showed that vaccine exposure to freezing temperatures is an ongoing issue even in wealthier countries [4] where detailed recommendations and guidelines exist [5–10]. Best practices to store vaccines at recommended temperatures require compliance with a number of structural and procedural aspects, ranging from refrigerator and thermometer equipment, temperature monitoring, storage procedures to the assignment of responsibilities [5,6,8–10]. In Germany, refrigerator management for laboratories and pharmacies is subject to quality management regulations [11], yet this is not the case for the primary care sector, where the majority of patients are vaccinated. Primary care is provided in privately owned general and pediatric practices who serve patients insured by statutory and private health insurance funds.

Many studies assessing vaccine storage practices rely on checklists, which are used as a self-administered tool or by third-party visual inspections combined with interviews [7,12–21]. These checklists are less prone to bias and are now considered standard procedures in quality management. The most frequently reported deficits from these studies were a lack of any type of thermometer (6.9% to 91.9%) [7,13–17,19], lack of a temperature logbook (26% to 94%) [13,15,19], lack of at least daily monitoring and recording (7.9% to 90.5%) [13,18], lack of regular thermometer checks (80%) [13,14], storing vaccines in door shelves (20.3% to 72.7%) [7,14,19], and storing items other than vaccines (3.6% to 96.3%) [7,12–15]. In 2014, an online-based survey among a random sample of German primary care physicians identified several vaccine storage deficits [22]. In response to the deficits, the Keep Cool study was initiated with the aim of ensuring good vaccine storage conditions. The Keep Cool intervention study consisted of three parts: after a baseline temperature survey of seven days (part one), general practices with temperature deficits were offered access to an online learning program (part two) [23] and were followed up seven weeks later [24]. This publication describes the baseline data of the Keep Cool study focusing on the quality of vaccine refrigerator management assessed in checklist-based direct observations of refrigerators.

## Material and methods

### Study design

The Keep Cool study is a prospective intervention study with two temperature measurement periods addressing vaccine cold chain management in primary care practices in Germany. The study protocol has been previously published [24]. The main objective is to improve vaccine storage conditions in German primary care practices. The primary outcome is the number of refrigerators with temperatures within the target range (2°C to 8°C) for seven days [23]. This manuscript presents the results for one of the secondary outcomes, namely the quality of vaccine refrigerator management as assessed by direct visual observations using a checklist. The primary outcome was the quality of vaccine refrigerator management based on reaching ten defined quality criteria. Ethical approval was obtained from the Ethic Commission of the Medical Faculty of the University of Duisburg-Essen (14-6118-BO). Participants provided written informed consent.

### Study population and recruitment procedure

The study invited all general practices of the teaching practice network associated with the University of Duisburg-Essen, Germany (N = 185). Practices (n = 17) involved in the study pre-tests (see 2.3.) were excluded, yielding a study population of 168 practices. Practices were recruited via email, fax and telephone. Practices not interested in participation received a short questionnaire via fax to allow for a non-participant analysis. Recruitment took place between January 2018 and August 2018.

### Development of the study instrument

As detailed guidelines or recommendations on vaccine storage are lacking in Germany, we reviewed recommendations and guidelines of comparable Western nations (United Kingdom, Australia, the United States Scotland, and Canada) to derive best practices for our setting [5,6,8–10]. Various structural and procedural aspects were identified and categorized into five core issues: 1) refrigerator, 2) temperature, 3) storage, 4) monitoring, and 5) responsibilities. For each core issue, quality criteria were derived from the literature. Individual aspects within these core issues that were included in several guidelines or relevant for the German setting were considered best practice. The criteria were subsequently used to develop a 10-item checklist which was compared and refined using checklists from eleven previous studies [7,12–19]. Items which were considered irrelevant for the German setting, e.g. storage in cool boxes, were not included. Because the checklists were embedded in the Keep Cool intervention study, items (e.g., designated responsibilities, frequency of resetting minimum-maximum thermometers, age of refrigerator) requiring enquiry beyond a visual inspection of the refrigerator were omitted to prevent an intervention bias. The instrument was finalized after a critical review by two general practice academics as regarded their relevance for the German primary care setting. In a pre-test with 21 refrigerators from 17 general practices of the network, we evaluated the feasibility of the study instrument and the practice visits. Data were collected by one of the research team members. Pre-testing lasted until data saturation occurred, i.e. no new aspects with regard to vaccine storage appeared. Answer keys were refined on the basis of the results of each pre-test. The final checklist consisted of 10 items and allowed for the recording of further observations (see Tables 1 and S1 for the original German version and S2 Table for the English translation). Based on scoring on the checklist items, a sum score was computed. We defined “good refrigerator management” as scoring positive on all 10 of 10 items.

**Table 1:** Quality indicator 'good refrigerator management' (10 items).

Item	Description	Governmental source
Refrigerator	1. Type of refrigerator suitable for vaccine storage	[6,8,10,25,26]
Temperature	2. Thermometer allows for digital minimum-maximum recording	[5,6,9,10]
	3. Temperature probe/thermometer in center of refrigerator	[6,8–10]
Monitoring	4. Temperature logbook visible near refrigerator	[5,6,8–10]
Storage	5. No vaccines stored on door shelves	[6,8–10]
	6. No food and no biomaterial stored	[5,6,8–10]
	7. All vaccines kept in original cardboard wrapping	[5,6,8–10]
	8. All vaccine boxes without contact to outer walls	[5,6,8–10]
	9. All vaccines in bins/baskets/separated shelves	[6,8,10]
	10. No overstocking (i.e., enough space between boxes)	[5,6,8–10]

### Data collection

Practice visits for data collection were conducted by the same researcher who had been involved in the pre-tests. The checklist was completed by means of a visual inspection while setting up data loggers to monitor the temperature for seven consecutive days. The checklist was completed immediately after each practice visit to prevent recall and intervention bias.

Temperatures were measured with a data logger (testo 175T, accuracy of ±0.4°C). We used continuous measurements over seven days at a logging interval of one reading every minute. According to standards [6,8,25,26], the data logger was positioned in the center of the refrigerator and placed in a plastic bin. The display was turned off and access to its memory was locked.

Data from the university teaching practice database were used to analyze associations between vaccine storage criteria and practice characteristics: type of practice (solo/group), number of practice team members differentiated by professional groups, practice size/caseload (patients per quarter), certified quality management, number of treatment rooms, thermometer in each vaccine refrigerator, vaccine spectrum offered, selected services offered (tropical medicine and/or yellow fever, travel medicine, adolescent preventive services, pediatric preventive services and/or adolescent medicine), and percentage of patients with statutory health insurance.

### Statistical analysis

To compare practices that participated in the study with those that did not (non-participants) for practice and physician characteristics, the χ^2^ test for categorical data and Student’s t-test for continuous data were used. Descriptive statistics were performed on an item level for all checklist items, including the quality criteria, and for the sum of quality criteria met. The latter was described for a) the total sample, b) refrigerators that continuously maintained the cold chain (2°C to 8°C), and c) refrigerators that reached critically low temperatures (≤0°C). For temperature data, readings of the 7-day monitoring period (10,080 minutes) were analyzed. The first 120 minutes after setting up each data logger were excluded from the analyses to allow the probe to acclimatize to the temperature of the refrigerator.

To analyze the association between practice characteristics and vaccine storage conditions, we used hierarchical generalized linear mixed models (GLMM) for binomial responses with random, practice-specific intercepts (to account for practices with more than one refrigerator). The sum score was dichotomized based on reaching more than half (6+) of the quality criteria. Independent characteristics were: type of practice (solo/group), number of patients in practice (≤1,750/ >1,750), percentage of patients with statutory health insurance, yellow fever license (yes/no), certified quality management (yes/no), and the provision of pediatric preventive services and/or adolescent medicine (yes/no).

Statistical analyses were performed using IBM SPSS Statistics for Windows, version 24 (Armonk, NY: IBM Corp.) and R, version 3.5.2. Percentages and mean values are reported for valid cases. Statistical significance was assigned at a level of p<0.05.

The trial is registered with the German Clinical Trials Register (DRKS00006561).

## Results

### Practice characteristics

Of the 168 practices invited, 64 agreed to participate (response rate: 38.1%). The non-participant analysis did not show differences regarding key characteristics, except that participants less frequently provided care to >1,750 patients per quarter (48.1% versus 68.1%, p = 0.029). See S3 Table for details.

Table 2 lists characteristics of participating practices. In total, 75 refrigerators were included in this study. 14.1% (n = 9) of the practices had more than one refrigerator for storing vaccines (eight practices had two refrigerators, one practice four refrigerators).

**Table 2:** Characteristics of participating practices (N = 64).

	n	%*
**Practice type, group**	38	59.4
**Mean no. of physicians in practice ± SD** (10)	2.1±1.2	
**Mean no. of medical assistants ± SD** (11)	5.3±3.3	
**Number of treatment rooms, ≤ 3** (10)	31	57.4
**Patients per practice per quarter (caseload), ≤ 1,750** (12)	27	51.9
**Percentage of patients with statutory health insurance, > 85%** (8)	37	66.1
**Certified quality management** (15)	14	28.6
**Physician qualifications (at least 1 physician in practice):**		
Travel medicine (10)	12	22.2
Tropical medicine and/or yellow fever license (10)	7	13.0
**Services offered** (10)		
Pediatric preventive services and/or adolescent medicine	22	40.7
Adolescent preventive services	44	81.5
**Practice vaccine spectrum** (10)		
Mean no. of vaccines ±SD	17.9±1.8	
Tetanus	54	100.0
Diphtheria	54	100.0
Pertussis	54	100.0
Influenza	54	100.0
Pneumococcal disease	54	100.0
Hepatitis A	54	100.0
Measles	54	100.0
Poliomyelitis	53	98.1
Hepatitis B	53	98.1
Tick-borne encephalitis	53	98.1
Rubella	53	98.1
Mumps	53	98.1
Meningococcal disease	52	96.3
Typhus	50	92.6
Varicella	50	92.6
Rabies	46	85.2
Human papilloma	44	81.5
Haemophilus influenzae B	41	75.9
Cholera	26	48.1
Rotavirus	14	25.9

*valid percentages

(missing values)

SD = standard deviation

### Description of refrigerator management

**Fig 1:**
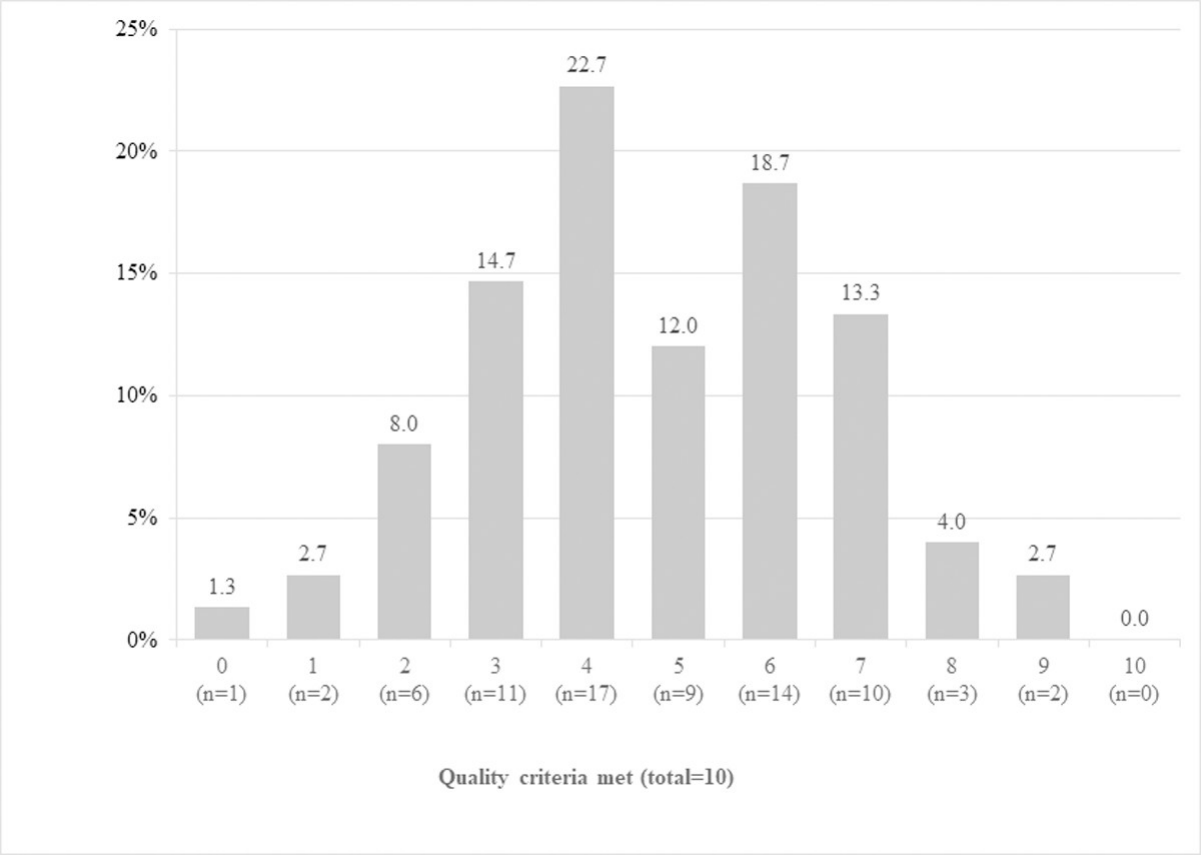
Frequencies for meeting the 10 quality criteria for 'good refrigerator management' (N = 75).

**Fig 2:**
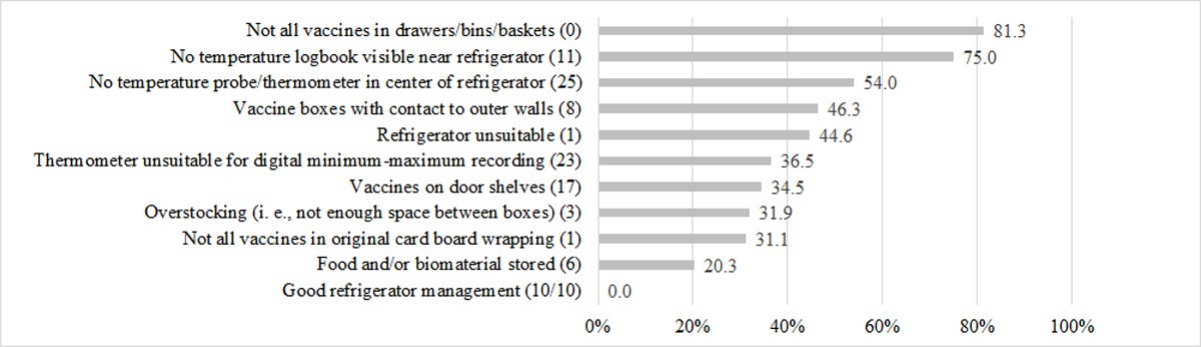
Frequencies for the 10 quality criteria for 'good refrigerator management' (N = 75). (missing values).

No practice had ‘good refrigerator management’, defined as meeting all ten quality criteria (see Fig 1). On average, refrigerators met 4.7 (SD = 1.9) criteria. See Fig 2 and Table 3 for an overview of the individual criteria. Refrigerators that continuously maintained the cold chain (n = 24) met 5.6 (SD = 2.3) items on average, and refrigerators with temperatures ≤0°C (n = 11) met 4.2 (SD = 1.4) items. Of all refrigerators that continuously maintained the cold chain, 62.5% (n = 15) met at least six of the quality criteria, compared to 27.5% (n = 14) of refrigerators with temperatures outside the cold chain (p = 0.008). At least six criteria were reached by 11.1% (n = 2) of refrigerators with temperatures <2°C and 18.2% (n = 2) of refrigerators that reached temperatures ≤0°C, respectively.

**Table 3:** Visual inspection of refrigerators used for vaccine storage based on (N = 75).

	n	%
**Type of refrigerator**		
Pharmaceutical grade*	9	12.0
Household refrigerator	66	88.0
Freezerless refrigerator*	30	45.5
Full-size dual-zone refrigerator/ freezer (separate exterior doors)*	2	3.0
Refrigerator with internal ice compartment	31	47.0
Refrigerator with internal non-insulated ice compartment	2	3.0
Household refrigerator, details unclear	1	1.5
**Functioning thermometer**		
None	20	26.7
1 functioning thermometer	53	70.7
2 functioning thermometers	2	2.7
**Type of functioning thermometer** (n = 57)		
Digital: Minimum-maximum+	23	40.4
Digital: Minimum-maximum remote+	3	5.3
Digital: Data logger cloud-based+	1	1.8
Digital: Integrated because pharmaceutical grade+	6	10.5
Digital: Digital thermometer, details unclear	4	7.0
Non-digital: Minimum-maximum	5	8.8
Non-digital: Plain thermometer without minimum-maximum function	15	26.3
**Placement of temperature probe/thermometer** (n = 57) (5)		
Close to outer walls	21	40.4
Center of refrigerator	17	32.7
In door shelves	8	15.4
Integrated (purpose-built refrigerator)	6	11.5
**Vaccines are stored in refrigerator door shelves** (17)	20	34.5
**Vaccine boxes are with contact to outer walls** (8)	31	46.3
**Vaccines NOT kept in original cardboard wrapping** (1)	23	31.1
Systematically unpacked	10	43.5
Sporadically unpacked	13	56.5
**Bins/baskets/separated shelves NOT used for all vaccines**	61	81.3
**Food and/or biomaterial stored** (8)	14	20.3
Food stored (1)	10	71.4
Biomaterial stored (8)	4	28.6
**Temperature logbook NOT visible in vicinity of refrigerator** (11)	48	75.0
**No. of entries in logbook** (62)		
Not daily	7	53.8
1x/day	6	46.2
2x/day	0	0.0

(missing values)

*suitable for vaccine storage according to NIST [25,26] and CDC [8].

+suitable for vaccine storage according to [5,6,9,10].

### Type of refrigerator used

Nine practices had purpose-built refrigerators for pharmaceuticals (12%), while the remaining used household refrigerators (n = 66; 88.0%) (Table 3). Of the household refrigerators, 50.8% (n = 33, n = 1 missing) were unsuitable for vaccine storage: 93.9% (n = 31) had an internal ice compartment with a less insulated separate door beyond the main exterior door, and two refrigerators had a non-insulated ice compartment without an extra door (6.1%).

### Type of thermometer used

Any type of functioning thermometer was absent in 26.7% (n = 20) of the refrigerators (see Table 3 for an overview of the thermometers used). Three thermometers were defective according to physicians or practice assistants, and one was placed next to the refrigerator. A thermometer that allowed for digital minimum-maximum recording or better was lacking in 36.5% (n = 19) of refrigerators. Two refrigerators were equipped with two thermometers each. Three thermometers allowed remote control and one was a data logger.

### Storage of food and biomaterial

Food or biomaterial was stored in 20.3% (n = 14) of the refrigerators alongside vaccines (Table 3). Of these, food was stored in 71.4% (n = 10), most frequently water, soft drinks, milk and bread spreads (meat and cheese). We also found sparkling wine, fresh cherries and yoghurt. One practice used half of the refrigerator’s volume for the storage of foodstuffs (inside plastic tubs without lids). Drinks were usually stored in the door.

### Placement of temperature probe/thermometer

In 54.0% (n = 27, 25 missings) of refrigerators a temperature probe/thermometer was not integrated or not placed in the center: 40.4% (n = 21) of the probes/thermometers had contact with external walls, 52.4% (n = 11) of these with the back wall. In six pharmaceutical-grade refrigerators, the probe was permanently installed. In one refrigerator, the thermometer was in the door shelf below the plastic container for eggs.

### Storage in original cardboard wrapping

In 31.1% (n = 23) of the refrigerators, not all vaccines were stored in their original cardboard wrapping (Table 3). Vaccines were systematically unpacked in ten refrigerators; most of the time this concerned the same types of vaccines (e.g. influenza). In six practices, vaccines were removed from their cardboard wrapping and stored in their plastic container.

### Temperature logbook near refrigerator

In 75.0% (n = 48) of cases, no temperature logbook was found near the refrigerator. No practice recorded the temperature twice daily; see Table 3 for details. Temperature logbooks varied in quality, with most of them indicating only the current temperature.

### Stocking of vaccines (overstocking, storage in door shelves, use of bins/baskets/separate shelves, contact of vaccine boxes with outer walls)

In 31.9% (n = 23) of cases, refrigerators were overstocked, i.e., not enough space was left between boxes or vaccines were removed from their cardboard wrapping to save space. In 34.5% (n = 20) of refrigerators, vaccines were stored in door shelves, in one practice even below the plastic container for eggs. One practice kept all vaccines in the door shelves, ‘reserving the main body for other things’. In 81.3% (n = 61) of refrigerators, bins/baskets or separate shelves were not used for all vaccines. Of these, 4.9% (n = 3) used containers only for some types of vaccines, and 11.5% (n = 7) only for all unwrapped vaccines. In 46.3% (n = 31) of refrigerators, vaccine cardboard boxes had contact with outer walls.

### Associations between quality criteria and practice characteristics

An analysis using GLMM showed no significant associations between scoring positive on at least half (6 out of 10) of the quality criteria and the independent variables considered (S4 Table).

## Discussion

Of the 75 refrigerators analyzed, none met all ten quality criteria for ‘good refrigerator management’. The items with the highest potential for improvement in at least 45% to 81% of refrigerators were using bins, baskets or other means to organize all vaccines, keeping a temperature logbook visible near the refrigerator, measuring temperatures in the center of the refrigerator, preventing contact of vaccine boxes with outer walls, and using refrigerator types suitable for vaccine storage.

Overall, the amount of avoidable vaccine management errors is remarkable. The types of errors are rather diverse ranging from structural aspects concerning equipment to procedural aspects such as unwrapping and overstocking vaccines. When the sum of additional observations (see S5 Table) is further taken into account, refrigerator management can be described as markedly heterogeneous between practices with an enormous need for improvement. A positive association between reaching more than six quality criteria and temperatures in the target range was identified. However, we did not identify associations between reaching more than six quality criteria and any practice characteristics. Interestingly, also physicians’ qualification for tropical medicine and/or yellow fever (special license required) was not associated with better quality.

When interpreting the results it must be noted that previous studies indicate that quality in vaccine storage is not constant but subject to fluctuations [13,27]. For instance, some of the deficits, e.g. unwrapping of vaccines or contact with outer wall, might be greater during the influenza season when practices store larger quantities of vaccines. The influenza season was not a factor in our study, which was conducted in spring and summer.

Comparing our data with other studies is difficult due to the heterogeneity of quality indicators and setting-specific characteristics (e.g., storage of frozen vaccines, which are used in our setting). We collected data on the use of tools to organize vaccines, the most frequently encountered deficit. Other studies focused more on organizational methods such as organizing by type of vaccines [15] and expiration dates [7,12,13,16], and less on tools that facilitate these methods. The identified absence of a temperature logbook is within the range of previous studies (26% to 94%; our study: 75.0%) [13,15,19]. However, actual numbers might be higher considering that our observations focused only on visible logbooks. Regarding the location of temperature-monitoring devices, a study in the US reported that 86.0% positioned their temperature-monitoring device not in the center but rather at the front of the refrigerator (51.2%) [28]. Actual contact of vaccine boxes with outer walls was not mentioned elsewhere. Regarding the type of refrigerator, ‘full-size dual-zone refrigerator/freezers (separate exterior doors)’ were used by 3% in our sample, compared to at least 44.4% of US physicians [16,28]. Pharmaceutical-grade refrigerators were used by 3.9% to 13.3% of Australian and US general practices, compared to 12.0% in our sample [28,29]. Smaller refrigerator/freezer units were used by 15.6% to 51.2% of US and Australian practices (53% in our study) [16,28,29], 36% of which lacked a separately sealed freezer compartment (5.7% in our study) [16]. In Italy, of 39 vaccination offices in 1999, 24% did not have any refrigerator and relied on neighboring facilities; three of seven practices selected for the monitoring used pharmaceutical-grade refrigerators [17]. The absence of any type of thermometer was reported in 6.9% to 91.9% of refrigerators [7,13–16,18,19,21]. In a study among US physicians, 37.8% also had a backup thermometer (2.7% in our study) [28]. Whether the thermometer was suitable for recording the digital minimum-maximum was considered in only one study (0% compared to 36.5% in our study) [14]. We know from other studies with self-administered questionnaires that unspecified minimum-maximum thermometers are lacking in 18.2% to 100% of refrigerators [17,20]. Storage of vaccines in door shelves was reported in 20.3% to 52.6% as compared to 34.5% in our study [7,13,19].

### Overstocking as a cause of most vaccine storage errors

Many of the vaccine storage errors identified in our study trace back to a mismatch between the storage capacity available in each practice refrigerator and the volume occupied by each vaccine (and diluent), including its cardboard wrapping. Practices that overstock engage in unacceptable procedures. Examples include 1) systematically storing vaccines without their insulating cardboard wrapping to save space, 2) using door shelves for storage, 3) allowing contact with external walls, 4) not using baskets/bins for the sake of organization (also regarding the opportunity to organize vaccines by their expiration date), 5) placing vaccines in refrigerators intended for the food of the practice team, and 6) failing to permanently install the probe/thermometer in the center of the refrigerator. The aspect of overstocking and its many consequences are discussed in guidelines, but have not been studied elsewhere. Practical advice includes a) improving the planning by reconciling the number of vaccinations administered, e.g. in a month, with according vaccine orders, b) reducing the number of vaccines stored at any given time by making use of pharmacy deliveries more frequently, c) investing in a larger refrigerator, d) refraining from food storage, and e) installing regular monitoring as well as f) supervisory checks.

### Strengths and limitations

The strength of this study lies in having obtained data by standardized, objective observations as opposed to self-reported methods. There are also several limitations. We designed the checklist as a measurement tool for an intervention study. To prevent bias, items that required direct questioning or closer inspection were excluded (e.g., responsibilities, frequency/procedure of resetting minimum-maximum thermometers, temperature logbooks not immediately visible). Collecting data by observation could have induced a measurement bias. First, the amount of items and the chaotic storage in the refrigerators and, based on that, the time needed to set up the monitoring device were the defining factors for the time that doors were left open. Most notes to complete the checklist had to be made within 3 to 20 seconds. Therefore, we allowed for the completion of missing items during the second practice visit after seven days when refrigerators were accessed again to download the data from the temperature loggers. Second, observations might be different at other times of the year [13,27]. Regarding the dependent variable, we selected meeting more than half of the ten quality criteria as the primary outcome, even though we consider meeting all criteria as important and assign an equal significance to the individual criteria. However, no practice met all ten criteria and the total number of refrigerators that met seven criteria or more was low. Participating practices are part of a teaching practice network. A potential selection bias can be excluded as we showed in a prior study that the practice sample in Essen is representative for general practices in Germany [30]. In order to assess participation bias, we conducted thorough non-responder analyses.

## Conclusion

Our results show that current storage conditions are a threat to the effectiveness of immunizations, our most effective public health strategy against contagious diseases. Efforts to guarantee quality-ensured cold chain management on a regulatory, educational and practice level are urgently needed. On a regulatory level, more detailed recommendations/guidelines/quality management regulations for primary care practices are needed, addressing e.g. acceptable types of refrigerators and thermometers. On an educational level, physicians and medical assistants need to be made aware of the relevance and procedures of adequate vaccine storage during their training. To assure widespread implementation of recommendations, an online-based learning program is currently being tested.

## Supporting information

S1 Table Checklist for the visual inspection of refrigerators (German version). (DOCX)

S2 Table Checklist for the visual inspection of refrigerators (English translation). (DOCX)

S3 Table Non-participant analysis (n = 168). (DOCX)

S4 Table Hierarchical generalized linear mixed models (GLMM) for associations between practice characteristics and reaching more than half (6+) of the ten quality criteria. (DOCX)

S5 Table Additional observations. (DOCX)
