# Supplementary material for: Visual inspection of vaccine storage conditions in general practices: A study of 75 vaccine refrigerators
Source: PLoS One. 2019 Dec 3;14(12):e0225764. doi: 10.1371/journal.pone.0225764 (PMC6890257; doi:10.1371/journal.pone.0225764)
Supplement: S1 Table — (DOCX) [file pone.0225764.s001.docx]

**S1 Table. Checklist for the visual inspection of refrigerators (German version).**

Thermometer ID: _________ (bei 2 Kühlschränken Zusatzinfo zum Raum: _________)

Praxis ID: _________

Visuelle Checkliste der Impfstofflagerung

| **Angaben zum Studienkühlschrank:** | |  | | |
| --- | --- | --- | --- | --- |
| 1. Art des Kühlschranks akzeptabel für Impfstofflagerung | | □^1^ Spezialkühlschrank__________________  □^2^ Haushaltsmodell, Details unklar | | |
|  | |  | □^21^ Mini KS: innenl. EF  □^22^ Mini KS: ohne EF  □^23^ Hüfthoher KS: innenl. EF  □^24^ Hüfthoher KS: ohne EF  □^25^ Mannshoher KS: innenl. EF  □^26^ Mannshoher KS: ohne EF  □^27^ Mannshohe Kühl-Gefrierkombi  □^28^ Hüfthoher KS: innenl. nicht isoliertes EF  □_____________________________ | |
|  | | □^3^ unklar__________________ | | |
| 1. Thermometer vorhanden | | □^2^ Nein □^3^ unklar__________________ | | |
|  | | □^1^ Ja | | |
|  | |  | | Thermometer 1:  □^1^ Min-Max  □^2^ Datenlogger  □^3^ Digital, unklar ob Min-Max  □^4^ Nicht digital (bitte zeichnen)  □^5^ unklar ____________________ |
|  | | □^4^ 2 Thermometer | | |
|  |  |  | | Thermometer 2:  □^1^ Min-Max  □^2^ Datenlogger  □^3^ Digital, unklar ob Min-Max  □^4^ Nicht digital (bitte zeichnen)  □^5^ unklar ____________________ |
| 1. Lage des Fühlers | | Thermometer 1: _________________ | | |
|  |  | Thermometer 2: _________________ | | |
| 1. Impfstoffe in der Tür | | □^1^ Ja □^2^ Nein □^3^ unklar _____________ | | |
| 1. Impfstoffe haben Kontakt mit den Außenwänden | | □^1^ Ja □^2^ Nein □^3^ unklar _____________ | | |
| 1. Impfstoffe in Originalverpackung | | □^1^ Ja □^2^ Nein, vereinzelt ausgepackt  □^3^ Nein, systemisch ausgepackt  □^4^ unklar _____________ | | |
| 1. Körbe/Fächer verwendet | | □^1^ Ja □^2^ Nein □^3^ zum Teil □^4^ unklar ____________ | | |
| 1. Separater Kühlschrank | | □^1^ Ja □^2^ Nein, Lebensmittel gelagert  □^3^ Nein, organische Produkte gelagert  □^4^ unklar _____________ | | |
| 1. Logbuch in Sichtweite | | □^2^ Nein □^3^ unklar _____________ | | |
|  | | □^1^ Ja | | |
|  | |  | | Anzahl Einträge im Logbuch  □^1^ 1x/Tag  □^2^ 2x/Tag  □^3^ nicht täglich  □^4^ unklar _____________ |
| 10. Subjektiv: zu hohe Füllung | | □^1^ Ja □^2^ Nein □^3^ unklar _____________ | | |
| 11. Was ist sonst noch aufgefallen: | | _____________________________________________ | | |
|  | Anzahl Kühlschränke, die für Impfstoffe verwendet werden | □^1^ 1 □^2^ 2 □^3^ 3+ □^4^ unklar | | |
|  | Medikamente gelagert | □^1^ Ja □^2^ Nein □^3^ unklar _____________ | | |
|  | Beschriftungen verwendet | □^1^ Ja □^2^ Nein □^3^ zum Teil □^4^ unklar ____________ | | |
|  | Gemüsefächer entfernt | □^1^ Ja □^2^ Nein □^3^ unklar _____________ | | |

EF=Eisfach
