# Supplementary material for: Visual inspection of vaccine storage conditions in general practices: A study of 75 vaccine refrigerators
Source: PLoS One. 2019 Dec 3;14(12):e0225764. doi: 10.1371/journal.pone.0225764 (PMC6890257; doi:10.1371/journal.pone.0225764)
Supplement: S2 Table — (DOCX) [file pone.0225764.s002.docx]

**S2 Table. Checklist for the visual inspection of refrigerators (English translation).**

Thermometer ID: _________ (in case of 2 refrigerators additional information on room: ________)

Praxis ID: _________

Visual Checklist for Vaccine Storage

| **Details of the study refrigerator:** | |  | | |
| --- | --- | --- | --- | --- |
| 1. Type of refrigerator acceptable for vaccine storage | | □^1^ Pharmaceutical grade__________________  □^2^ Household refrigerator | | |
|  | |  | □^21^ Small: internal ice compartment  □^22^Small: without ice compartment  □^23^ Waist-high: internal ice compartment  □^24^ Waist-high: Freezerless  □^25^ Full-size: internal ice compartment  □^26^ Full-size: Freezerless refrigerator  □^27^ Full-size dual-zone refrigerator/freezer  □^28^ Waist-high: internal non-insulated ice compartment  □_____________________________ | |
|  | | □^3^ Unclear__________________ | | |
| 1. Thermometer available | | □^2^ No □^3^ Unclear__________________ | | |
|  | | □^1^ Yes | | |
|  | |  | | Thermometer 1:  □^1^ Min-Max  □^2^ Data logger  □^3^ Digital, unclear whether Min-Max  □^4^ Non-digital (please draw)  □^5^ Unclear ____________________ |
|  | | □^4^ 2 Thermometer | | |
|  |  |  | | Thermometer 2:  □^1^ Min-Max  □^2^ Data logger  □^3^ Digital, unclear whether Min-Max  □^4^ Non-digital (please draw)  □^5^ Unclear ____________________ |
| 2. Placement of temperature probe/thermometer | | Thermometer 1: _________________ | | |
|  |  | Thermometer 2: _________________ | | |
| 1. Vaccines in door shelves | | □^1^ Yes □^2^ No □^3^ Unclear _____________ | | |
| 1. Vaccines with contact to outer walls | | □^1^ Yes □^2^ No □^3^ Unclear _____________ | | |
| 1. Vaccines in original cardboard wrapping | | □^1^ Yes □^2^ No, sporadically unpacked  □^3^ No, systematically unpacked  □^4^ Unclear _____________ | | |
| 1. Bins/baskets used | | □^1^ Yes □^2^ No □^3^ Partly □^4^ Unclear ____________ | | |
| 1. Separate refrigerator | | □^1^ Yes □^2^ No, food stored  □^3^ No, biomaterials stored  □^4^ Unclear _____________ | | |
| 1. Logbook visible in vicinity | | □^2^ No □^3^ Unclear _____________ | | |
|  | | □^1^ Yes | | |
|  | |  | | No. of entries in logbook  □^1^ 1x/day  □^2^ 2x/day  □^3^ Not daily  □^4^ Unclear _____________ |
| 10. Subjective: overstocked | | □^1^ Yes □^2^ No □^3^ Unclear _____________ | | |
| 11. Other aspects noticed: | | _____________________________________________ | | |
|  | No. refrigerators used for vaccine storage | □^1^ 1 □^2^ 2 □^3^ 3+ □^4^ Unclear | | |
|  | Medications stored | □^1^ Yes □^2^ No □^3^ Unclear _____________ | | |
|  | Labelling used | □^1^ Yes □^2^ No □^3^ Partly □^4^ Unclear ____________ | | |
|  | Vegetable bins removed | □^1^ Yes □^2^ No □^3^ Unclear _____________ | | |
