# Supplementary material for: Visual inspection of vaccine storage conditions in general practices: A study of 75 vaccine refrigerators
Source: PLoS One. 2019 Dec 3;14(12):e0225764. doi: 10.1371/journal.pone.0225764 (PMC6890257; doi:10.1371/journal.pone.0225764)
Supplement: S4 Table — (DOCX) [file pone.0225764.s004.docx]

**S4 Table. Hierarchical generalized linear mixed models (GLMM) for associations between practice characteristics and reaching more than half (6+) of the ten quality criteria.**

|  |  |  |  | n | Estimate (beta) | SE | OR | 95% CI (OR) | p-value |
| --- | --- | --- | --- | --- | --- | --- | --- | --- | --- |
| **Practice type** | | | |  |  |  |  |  |  |
|  | Solo | | | 75 | -0.70 | 0.82 | 0.49 | 0.10-2.45 | 0.388 |
|  | Group (ref) | | |  |  |  |  |  |  |
| **Patients per practice per quarter (caseload)** | | | |  |  |  |  |  |  |
|  | ≤ 1,750 | | | 60 | -0.67 | 1.07 | 0.51 | 0.06-4.15 | 0.531 |
|  | > 1,750 (ref) | | |  |  |  |  |  |  |
| **Percentage of patients with statutory health insurance** | | | |  |  |  |  |  |  |
|  | ≤ 85% | | | 62 | -0.26 | 0.61 | 0.77 | 0.23-2.53 | 0.666 |
|  | > 85% (ref) | | |  |  |  |  |  |  |
| **Tropical medicine and/or yellow fever license** | | | |  |  |  |  |  |  |
|  | Yes | | | 62 | 1.97 | 1.75 | 7.14 | 0.23-222.25 | 0.262 |
|  | No (ref) | | |  |  |  |  |  |  |
| **Certified quality management** | | | |  |  |  |  |  |  |
|  | Yes | | | 56 | 2.38 | 1.64 | 10.8 | 0.44-266.70 | 0.146 |
|  | No (ref) | | |  |  |  |  |  |  |
| **Pediatric preventive services and/or adolescent medicine** | | | |  |  |  |  |  |  |
|  | Yes | | | 62 | -0.12 | 0.94 | 0.89 | 0.14-5.62 | 0.902 |
|  | No (ref) | | |  |  |  |  |  |  |
