# Supplementary material for: Visual inspection of vaccine storage conditions in general practices: A study of 75 vaccine refrigerators
Source: PLoS One. 2019 Dec 3;14(12):e0225764. doi: 10.1371/journal.pone.0225764 (PMC6890257; doi:10.1371/journal.pone.0225764)
Supplement: S5 Table — (DOCX) [file pone.0225764.s005.docx]

**S5 Table. Additional observations.**

| **Type of refrigerator** |
| --- |
| • Regarding storage capacity, 77.3% (n=58) of the refrigerators were waist-high or shorter, all others were taller. |
| • n=3 used the refrigerators as a place for the practice team members to store food. |
| • n=2 had separate refrigerators for different physicians, and one practice shared a refrigerator with a urology practice (specific shelves labelled accordingly). |
| **Thermometer** |
| • n=1 bought a new remote minimum-maximum thermometer after being contacted about the study participation for the first time; another practice bought two thermometers during the 7-day baseline monitoring period. |
| • A display outside the refrigerator was installed on three refrigerators. A further 10 thermometers were equipped with a cable and could have been set up accordingly. See Fig 3 for the most frequently encountered non-digital thermometers.   \| 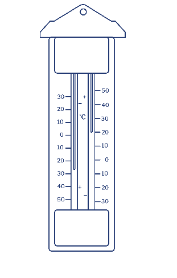 \| 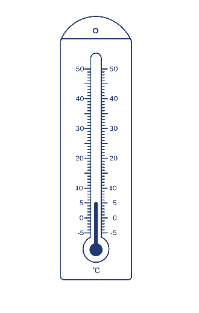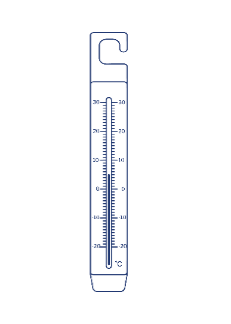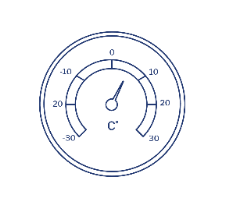 \| \| --- \| --- \| \| Non-digital minimum-maximum thermometer (n=6) \| Non-digital plain thermometer (n=9) \|   Fig 3. Common non-digital thermometers used. |
| • Four practices temperature-buffered the probes: one used a purpose-built metal block and another placed the probe in a cardboard box. Two had self-made constructions: one placed the probe in a closed urine cup filled with water; one used closed containers with glycol in two refrigerators. All other practices used probes that reflect the ambient air temperature. |
| **Logbook** |
| • In one practice, medical assistants considered their documented temperatures of 0 to 4°C to be acceptable, as they thought it was better for vaccines to be stored too cold. One practice was aware of their defective thermometer and kept a logbook where they always subtracted 3°C. |
| **Cardboard wrapping of vaccines** |
| • Where labels showing details of the batch number, dose etc. did not stick to the vials, labels were frequently stored separately from the vial, e.g. in door shelves. |
| • In several cases, single unwrapped vials were stored on steel (in a kidney dish), or had direct contact with glass/steel shelves/grids. |
| • In two refrigerators, practices stored individual unwrapped glass vials wrapped in a piece of paper secured with a rubber band. |
| • In many practices, boxes were not closed properly or the lids had been torn off, exposing the glass vials to ambient air. |
| **Use of bins/baskets** |
| • The bins/baskets used were made from cardboard, colored food boxes, plastic towers with drawers, or plastic containers with separate lids. One practice systematically stored unwrapped vaccines and insulin like toothbrushes in labelled plastic drinking cups. |
| **Contact with outer walls** |
| • In one practice, the cardboard wrappings were misshaped because of contact with side walls and due to on-and-off freezing and thawing. |
| • In one practice, an ice layer of 3 cm covered the back wall. |
| **Labels used for all vaccines/bins/baskets/drawers/shelves** |
| • Labels used for all vaccines/bins/baskets/drawers/shelves were used by 19.2% (n=14, 2 missing datasets). |
| **Miscellaneous** |
| • As part of the study, we placed a plastic bin with a data logger inside each refrigerator for seven days. In one practice we found a dead fly in the bin, another bin was filled with 2 mm of water, as we had to place it underneath the ice compartment in order to place it in the center. |
| • One practice reported having received a delivery packed in dry ice; this decreased the temperature to -0.4°C (not part of our 7-day baseline measurement). |
| • In one practice, the pharmacy had checked the refrigerator and placed a note on top of the refrigerator stating ‘not below 2°C’. |
| • 66.7% (n=42, 12 missing datasets) stored medicines in the refrigerator (insulin, Clexane). |
